# A human-derived Pseudorabies virus isolate induces Cav2.2-sensitive Ca^2+^ influx and JNK-associated retinal epithelial injury

**DOI:** 10.1128/spectrum.01655-26

**Published:** 2026-07-16

**Authors:** Chuyue Zhou, Hang Li, Lang Tian, Hao Zhang, Ruicheng Yang, Huanchun Chen, Xiangru Wang

**Affiliations:** 1 National Key Laboratory of Agricultural Microbiology, College of Veterinary Medicine, Huazhong Agricultural University 124443, Wuhan, China; 2 Key Laboratory of Preventive Veterinary Medicine in Hubei Province, the Cooperative Innovation Center for Sustainable Pig Production, Wuhan, China; 3 Engineering Research Center of Animal Biopharmaceuticals, Ministry of Education of the People’s Republic of China (MOE) 12543, Wuhan, China; 4 Frontiers Science Center for Animal Breeding and Sustainable Production, Wuhan, China; Changchun Veterinary Research Institute, Changchun, China

**Keywords:** Pseudorabies virus, ARPE-19, retinal epithelial injury, Ca^2+ ^influx, Cav2.2, glycoprotein K

## Abstract

Pseudorabies virus (PRV), the etiological agent of Aujeszky’s disease, remains a major threat to the swine industry. Several human PRV infections have recently been reported, including cases with severe ocular involvement, but how human-derived PRV isolates injure retinal cells remains poorly understood. Here, we used the human-derived PRV isolate hSD-1/2019 and ARPE-19 cells to define an injury-associated Ca^2+^ signaling pathway in retinal epithelial cells. hSD-1/2019 infection induced pronounced extracellular Ca^2+^ influx, CaMKII and JNK1/2 activation, mitochondrial dysfunction, and membrane-compromising cellular injury. Pharmacological screening identified a prominent Cav2.2-sensitive component in the infection-associated Ca^2+^ influx. Blockade of this component attenuated JNK1/2 activation, mitochondrial injury, and loss of membrane integrity, linking extracellular Ca^2+^ entry to downstream epithelial injury. In addition, PRV glycoprotein K (gK) expression promoted Cav2.2-sensitive Ca^2+^ elevation and JNK1/2 activation in transfected cells, supporting gK as a candidate upstream viral contributor to this response. Together, these findings suggest that hSD-1/2019 converts infection into a Ca^2+^ influx-driven injury program in retinal epithelial cells and identify a Cav2.2-sensitive Ca^2+^/JNK axis as a focused mechanism of PRV-associated retinal epithelial injury.

**IMPORTANCE:**

Pseudorabies virus (PRV) has long been considered primarily an animal pathogen, but recent human infections with severe ocular disease have raised concerns about its ability to damage human retinal cells. This study shows that the human-derived PRV isolate hSD-1/2019 engages extracellular Ca^2+^ influx to drive a JNK1/2-linked mitochondrial injury response in ARPE-19 cells. A Cav2.2-sensitive Ca^2+^ entry component was functionally associated with this process, and PRV gK was identified as a candidate viral contributor capable of engaging the Ca^2+^/JNK response. These findings provide a mechanistic framework for PRV-associated retinal epithelial injury and support further investigation in primary retinal cells and *in vivo* models.

## INTRODUCTION

Pseudorabies virus (PRV), also known as Suid herpesvirus 1, is an enveloped double-stranded DNA virus belonging to the subfamily *Alphaherpesvirinae* (1, 2). Other prominent members of this subfamily include herpes simplex virus types 1 and 2 (HSV-1, HSV-2), varicella-zoster virus, and bovine herpesvirus 1 (3). The mature PRV virion consists of a DNA-containing capsid, a proteinaceous tegument, and an outer envelope studded with at least 11 glycoproteins (gB, gC, gD, gE, gG, gH, gI, gK, gL, gM, and gN), which play crucial roles in viral entry, spread, and pathogenesis (4). Swine are the only natural reservoir for PRV, but its potential as a zoonotic pathogen has attracted increasing attention (5). Since 2017, several suspected human PRV infections have been reported in China, often in association with recently emerged PRV variant/class II genomic backgrounds (1, 6–8). Among these cases, severe ocular manifestations, including endophthalmitis, retinal detachment, and retinal necrosis, have been described (7–9). These observations raise an important question: how do human-derived PRV isolates injure retinal cells?

Retinal injury caused by viral infection is not only a consequence of viral replication but also reflects how infected cells respond to stress. Loss of plasma membrane integrity is particularly relevant in severe tissue injury because it can promote the release of intracellular danger signals, inflammatory responses, and irreversible cellular damage (10–13). Therefore, defining the signaling events that connect PRV infection to membrane-compromising retinal epithelial injury may help clarify how human-derived PRV isolates damage ocular tissues.

Calcium (Ca^2+^) is a central second messenger that links external stimuli to cellular stress responses (14). Cytosolic Ca^2+^ levels are tightly controlled by plasma membrane channels, intracellular stores, pumps, and exchangers (15, 16). During viral infection, disruption of Ca^2+^ homeostasis can reshape host signaling, influence viral replication, and promote cellular injury (17, 18). In neurotropic alphaherpesvirus infections, altered Ca^2+^ handling has been linked to neuronal dysfunction, viral transport, membrane remodeling, and pathogenesis (19–21). Because the extracellular Ca^2+^ concentration is much higher than the basal cytosolic Ca^2+^ concentration, sustained Ca^2+^ influx may represent an early and amplifying event in virus-induced injury (18). However, whether human-derived PRV infection engages extracellular Ca^2+^ entry during retinal epithelial injury remains unclear.

Sustained Ca^2+^ elevation can activate Ca^2+^-responsive kinases and stress signaling pathways. Ca^2+^/calmodulin-dependent protein kinase II (CaMKII) is a major sensor of intracellular Ca^2+^ changes and can connect Ca^2+^ overload to oxidative stress, mitochondrial dysfunction, and mitogen-activated protein kinase signaling (MAPK) (22, 23). c-Jun N-terminal kinase (JNK) is frequently activated during cellular stress and has been implicated in mitochondrial injury, inflammatory signaling, and loss of cell viability (24–26). Mitochondria are central hubs for integrating these injury signals (27), and mitochondrial dysfunction is commonly characterized by increased reactive oxygen species (ROS), dissipation of mitochondrial membrane potential (ΔΨm), and ATP depletion (28, 29). Whether PRV-induced Ca^2+^ influx is coupled to CaMKII/JNK activation and mitochondrial dysfunction in retinal epithelial cells has not been defined.

The retinal pigment epithelium (RPE) is essential for retinal barrier function, photoreceptor support, and local inflammatory homeostasis (30). The human retinal pigment epithelial cell line ARPE-19 has been widely used as an *in vitro* model to study RPE biology, retinal injury, and virus-host interactions (31–33). In this study, we used ARPE-19 cells and the human-derived PRV isolate hSD-1/2019 to investigate how PRV infection induces retinal epithelial cell injury. We focused on extracellular Ca^2+^ influx, Cav2.2-sensitive Ca^2+^ entry, CaMKII/JNK signaling, and mitochondrial dysfunction as interconnected events in this process, with the classical Ea strain included as a reference strain where appropriate. We further performed a candidate viral protein screen to explore viral contributors that may connect PRV membrane-associated functions to Ca^2+^ influx.

## RESULTS

### ARPE-19 cells support hSD-1/2019 infection under the experimental conditions

**Fig 1:**
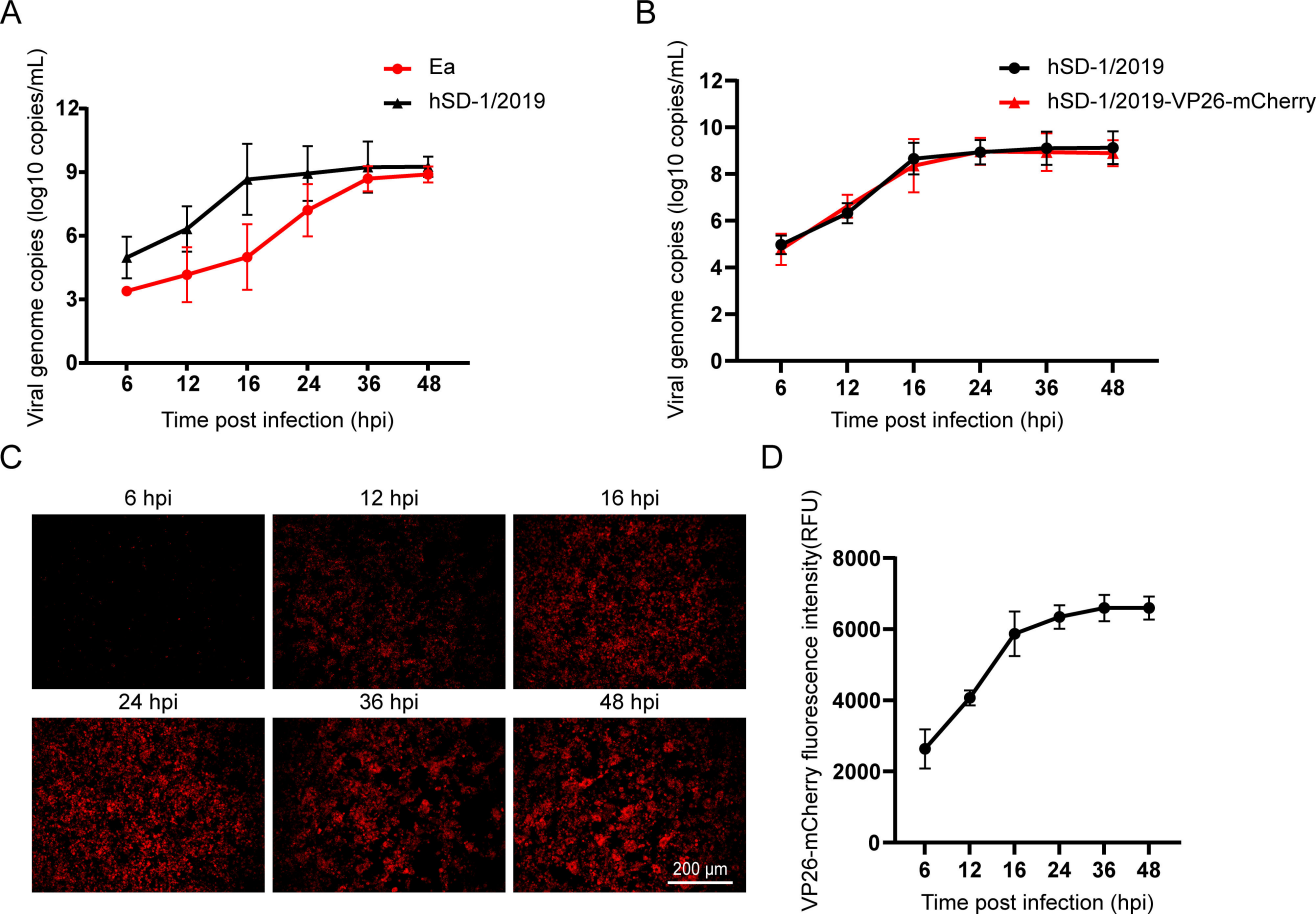
ARPE-19 cells support hSD-1/2019 infection under the experimental conditions. (**A**) Growth kinetics of the classical PRV strain Ea and the human-derived PRV strain hSD-1/2019 in ARPE-19 cells infected at MOI = 0.5. Total viral genome copies were quantified by qPCR at the indicated time points post-infection. (**B**) Growth kinetics of parental hSD-1/2019 and the VP26-mCherry reporter virus derived from hSD-1/2019 in ARPE-19 cells infected at MOI = 0.5. Viral genome copies were quantified by qPCR. (**C**) Representative fluorescence microscopy images showing VP26-mCherry signal in ARPE-19 cells infected with hSD-1/2019-VP26-mCherry at the indicated time points. Scale bar, 200 μm. (**D**) Quantification of VP26-mCherry fluorescence intensity in infected ARPE-19 cells over time. Data are presented as mean ± SD from three independent experiments.

We first examined PRV infection in ARPE-19 cells under the experimental conditions used in this study. At an MOI of 0.5, viral genome copies increased over time in both hSD-1/2019- and Ea-infected ARPE-19 cells (Fig. 1A). To monitor infection progression, we used a VP26-mCherry reporter virus derived from hSD-1/2019. The reporter virus showed genome-copy kinetics comparable to the parental virus (Fig. 1B), and VP26-mCherry fluorescence progressively increased in infected ARPE-19 cells over time (Fig. 1C and D). These results confirmed that ARPE-19 cells support hSD-1/2019 infection under the conditions used for subsequent injury and signaling analyses.

### hSD-1/2019 infection induces cytopathic retinal epithelial injury with increased membrane permeability

**Fig 2:**
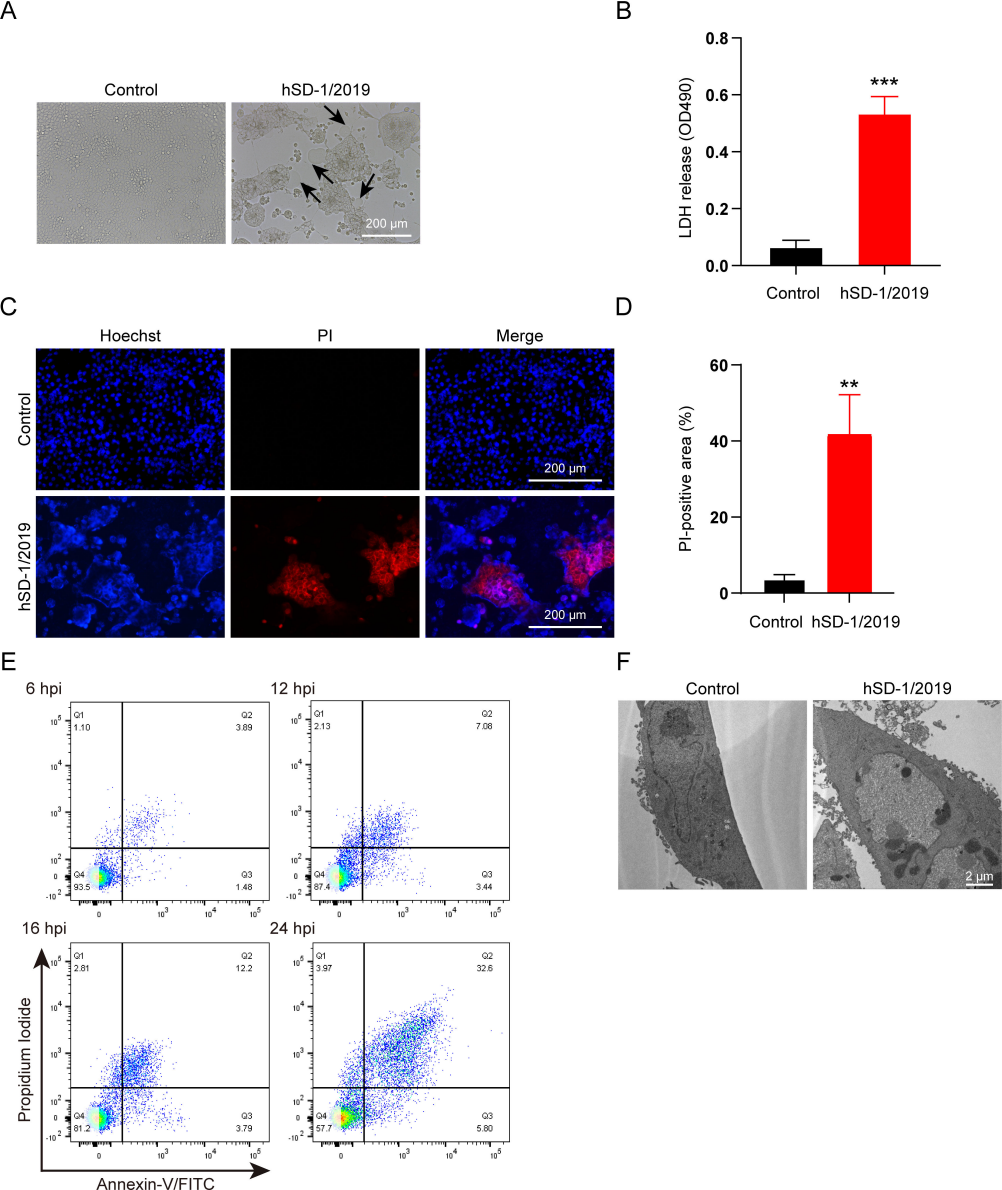
hSD-1/2019 infection induces cytopathic retinal epithelial injury with increased membrane permeability. ARPE-19 cells were infected with hSD-1/2019 at MOI = 0.5. (**A**) Phase-contrast microscopy showing CPE in infected ARPE-19 cells at 16 hpi. Arrows indicate swollen, detached, or aggregated cells. Scale bar, 200 μm. (**B**) Cytotoxicity in infected ARPE-19 cells at 16 hpi, assessed by LDH release assay (OD490 nm). (**C**) Representative fluorescence microscopy images of infected cells co-stained with PI and Hoechst 33342 at 16 hpi. Scale bar, 200 μm. (**D**) Quantification of PI-positive area following infection. (**E**) Flow cytometry analysis of Annexin V-FITC/PI-stained ARPE-19 cells at the indicated time points post-infection. (**F**) Transmission electron microscopy (TEM) of control and hSD-1/2019-infected ARPE-19 cells. Scale bar, 2 μm. Data are presented as mean ± SD from three independent experiments. Statistical significance was determined using a two-tailed Student’s *t*-test (**P < 0.01; ***P < 0.001).

We next examined the cellular injury phenotype induced by hSD-1/2019 infection. At 16 h post-infection (hpi), infected ARPE-19 cells showed obvious cytopathic effects (CPE), including cell rounding, swelling, detachment, and formation of large cell aggregates or syncytium-like structures (Fig. 2A). This was accompanied by a marked increase in lactate dehydrogenase (LDH) release, indicating increased cytotoxicity and loss of membrane integrity (Fig. 2B).

Propidium iodide (PI)/Hoechst staining showed broad PI-positive signals in infected cells (Fig. 2C). Quantitative analysis confirmed a significant increase in PI-positive area following infection (Fig. 2D). Annexin V/PI flow cytometry further showed that infected cells progressively shifted toward PI-positive populations over time, especially at 16 and 24 hpi (Fig. 2E). Transmission electron microscopy (TEM) analysis revealed chromatin marginalization in infected cells (Fig. 2F). Together, these results show that hSD-1/2019 infection causes cytopathic retinal epithelial cell injury characterized by increased membrane permeability and cytotoxicity.

### hSD-1/2019-induced injury depends on extracellular Ca^2+^ influx

**Fig 3:**
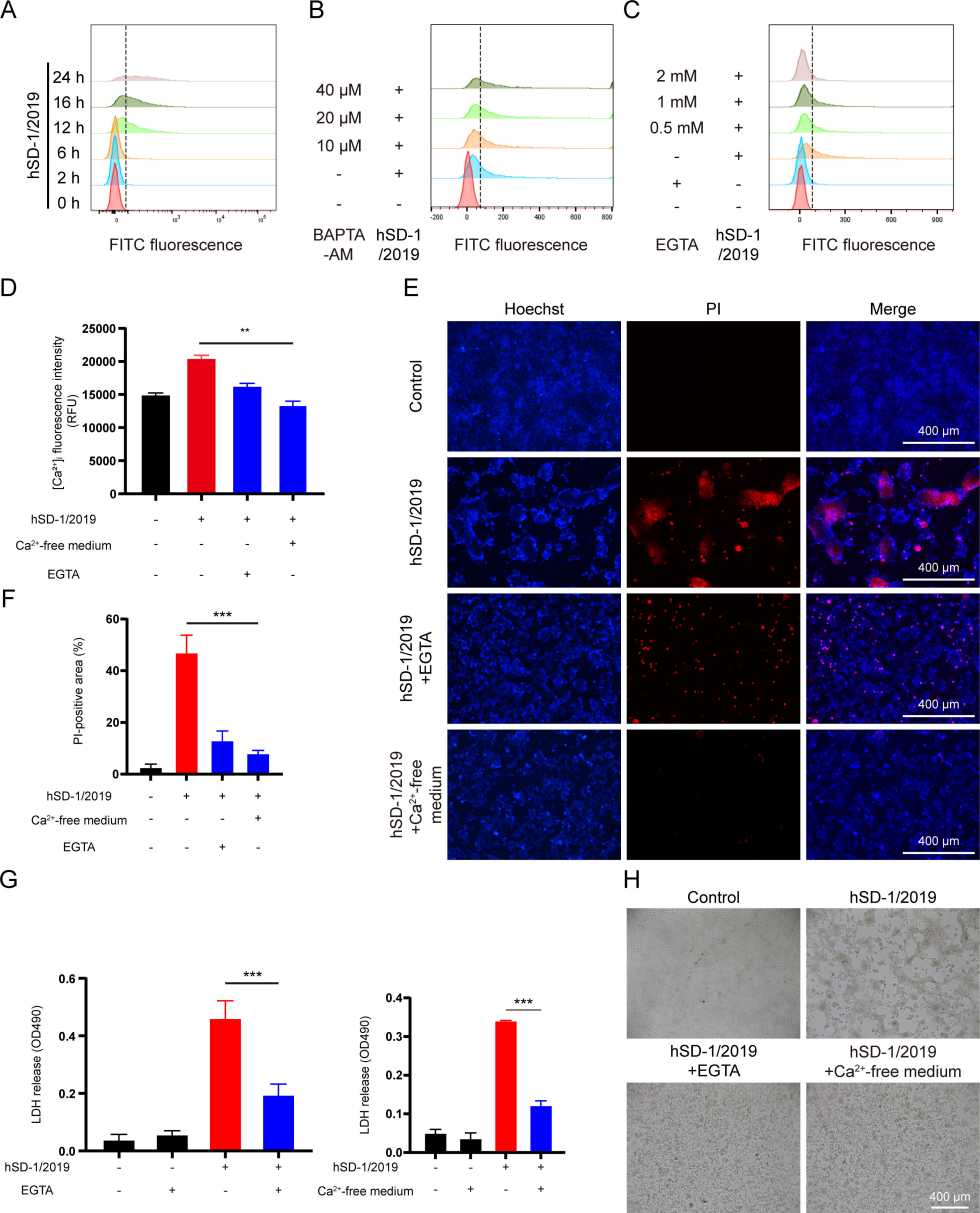
hSD-1/2019-induced injury depends on extracellular Ca^2+^ influx. ARPE-19 cells were infected with hSD-1/2019 at MOI = 0.5, and intracellular Ca^2+^ concentration ([Ca^2+^]ᵢ) was measured using Fluo-3/AM. (**A**) Time-course analysis of [Ca^2+^]ᵢ in infected ARPE-19 cells, measured by flow cytometry. (**B, C**) [Ca^2+^]ᵢ in infected cells at 16 hpi following pretreatment with the intracellular Ca^2+^ chelator BAPTA-AM or the extracellular Ca^2+^ chelator EGTA at the indicated concentrations. (**D**) Quantification of [Ca²^+^]ᵢ in infected cells treated with EGTA (2 mM) or cultured in Ca^2+^-free medium, measured by Fluo-3/AM fluorescence intensity. (**E**) PI/Hoechst staining of infected cells treated with EGTA (2 mM) or cultured in Ca^2+^-free medium. Scale bar, 400 μm. (**F**) Quantification of PI-positive area. (**G**) LDH release in infected cells treated with EGTA (2 mM) or cultured in Ca^2+^-free medium. (**H**) Phase-contrast microscopy showing CPE in infected cells treated as indicated. Scale bar, 400 μm. Data are presented as mean ± SD from three independent experiments. Statistical significance was determined using a two-tailed Student’s *t*-test (**P < 0.01; ***P < 0.001).

We next examined whether hSD-1/2019 infection altered intracellular Ca^2+^ concentration ([Ca^2+^]ᵢ) in ARPE-19 cells. Using Fluo-3/AM staining, we observed a time-dependent increase in [Ca^2+^]ᵢ following hSD-1/2019 infection (Fig. 3A). Treatment with the intracellular Ca^2+^ chelator BAPTA-AM had a limited effect on this increase, whereas the extracellular Ca^2+^ chelator EGTA reduced [Ca^2+^]ᵢ in a dose-dependent manner (Fig. 3B and C). Consistently, EGTA treatment or culture in Ca^2+^-free medium markedly reduced [Ca^2+^]ᵢ in infected cells (Fig. 3D), supporting extracellular Ca^2+^ influx as the main source of the infection-associated Ca^2+^ increase.

We then assessed whether extracellular Ca^2+^ influx was linked to the injury phenotype. Both EGTA treatment and Ca^2+^-free medium reduced PI staining, PI-positive area, LDH release, and infection-induced CPE (Fig. 3E through H). Viral genome accumulation and VP26-mCherry fluorescence were not markedly reduced under these conditions (Fig. S1A through C). Together, these results indicate that hSD-1/2019 infection induces extracellular Ca^2+^ influx that is functionally linked to cytopathic retinal epithelial injury.

### JNK1/2 activation is associated with hSD-1/2019-induced retinal epithelial injury

**Fig 4:**
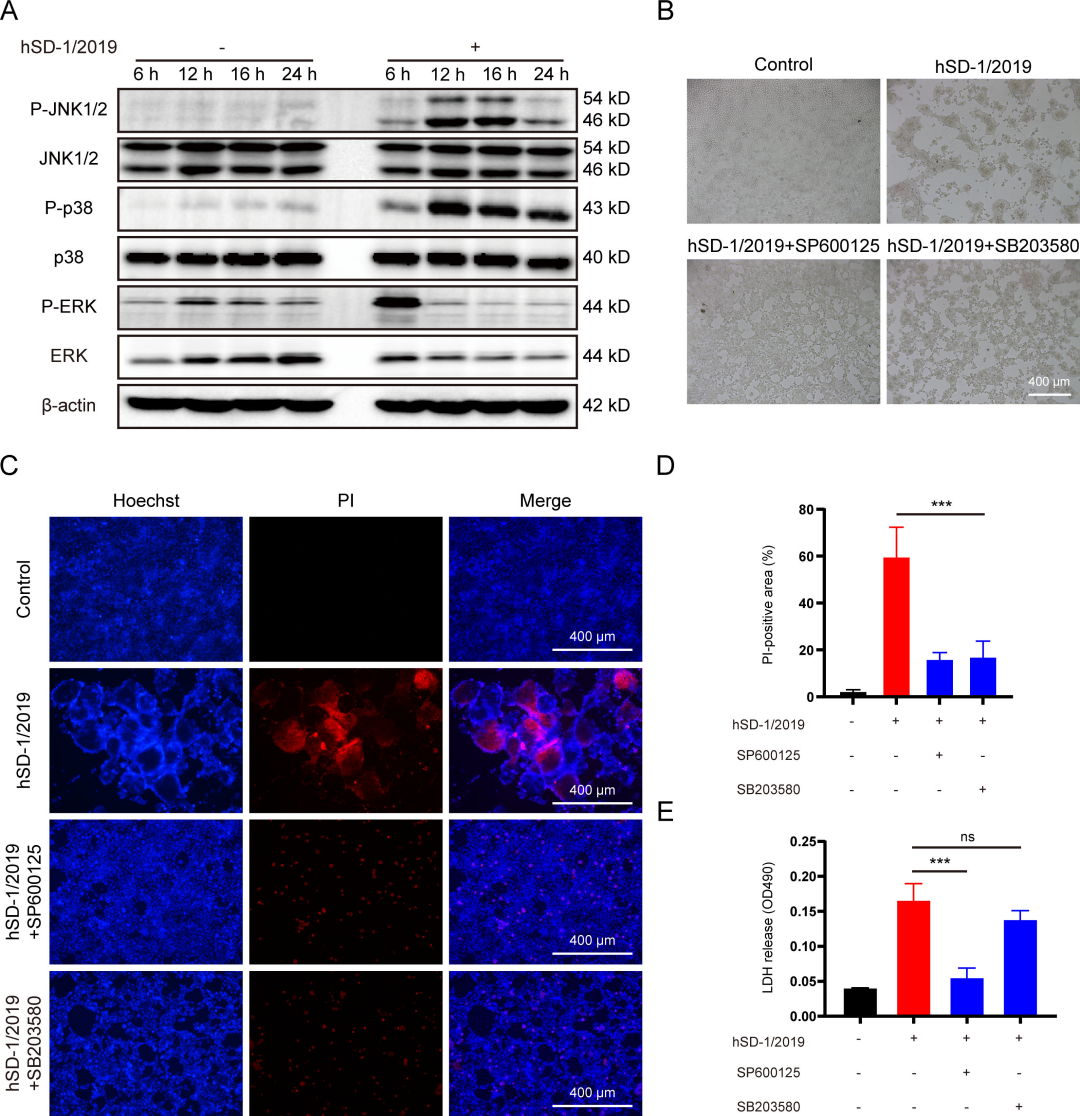
JNK1/2 activation is associated with hSD-1/2019-induced retinal epithelial injury. (**A**) Western blot analysis of the phosphorylation levels of ERK1/2, JNK1/2, and p38 in ARPE-19 cells infected with hSD-1/2019 (MOI = 0.5) at the indicated times post-infection. Total ERK1/2, JNK1/2, and p38 were used as corresponding controls, and β-actin served as the loading control. (**B**) CPE (scale bar, 400 μm), (**C**) PI/Hoechst staining (scale bar, 400 μm), (**D**) quantification of PI-positive area, and (**E**) LDH release in infected cells at 16 hpi following pretreatment with the JNK1/2 inhibitor SP600125 (10 μM) or the p38 inhibitor SB203580 (10 μM). Data are presented as mean ± SD from three independent experiments. Statistical significance was determined using a two-tailed Student’s *t*-test (***P < 0.001; ns, not significant).

MAPK signaling pathways are commonly engaged during viral infection and can influence cellular stress responses and injury-related signaling (34–36). We therefore examined MAPK activation in hSD-1/2019-infected ARPE-19 cells. Western blot analysis revealed sustained phosphorylation of JNK1/2 and p38, whereas ERK1/2 phosphorylation was not markedly increased under the same conditions (Fig. 4A).

To assess the functional relevance of these pathways, ARPE-19 cells were pretreated with the JNK1/2 inhibitor SP600125 or the p38 inhibitor SB203580 before infection. Both inhibitors reduced PI-positive staining and PI-positive area to some extent (Fig. 4C and D). However, SP600125 showed a more consistent protective effect, as reflected by reduced CPE and LDH release, whereas SB203580 had a weaker effect on LDH release (Fig. 4B and E). Viral genome accumulation and VP26-mCherry fluorescence were not markedly reduced by SP600125 under the same conditions (Fig. S1D through F). These results suggest that JNK1/2 activation is involved in hSD-1/2019-induced retinal epithelial cell injury.

### CaMKII inhibition attenuates hSD-1/2019-induced retinal epithelial cell injury

**Fig 5:**
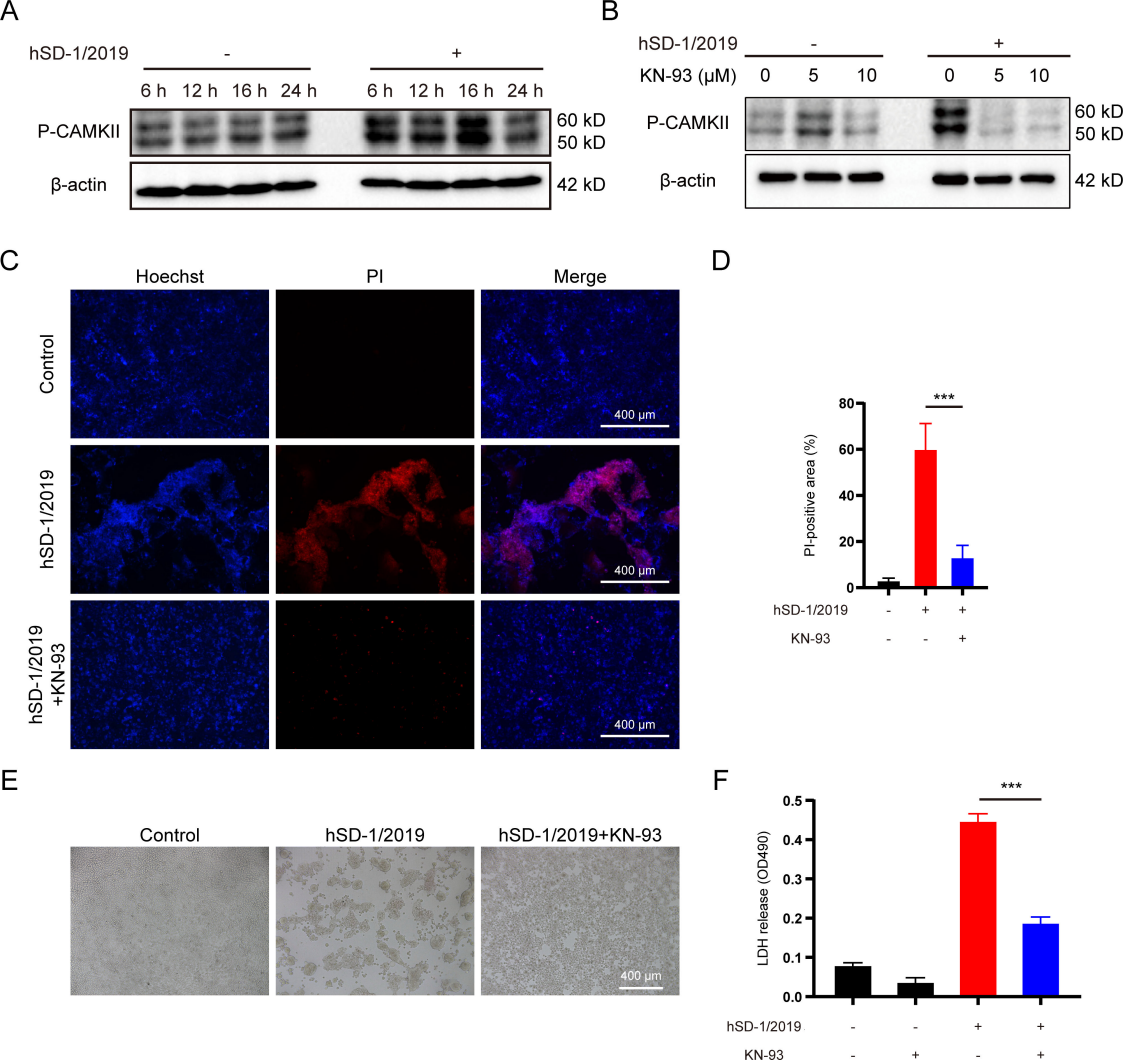
CaMKII inhibition attenuates hSD-1/2019-induced retinal epithelial cell injury. (**A**) Western blot analysis of phospho-CaMKII levels in ARPE-19 cells infected with hSD-1/2019 (MOI = 0.5) for the indicated times. β-actin served as the loading control. (**B**) Western blot analysis of phospho-CaMKII levels in infected cells treated with the CaMKII inhibitor KN-93 at the indicated concentrations. β-actin served as the loading control. (**C**) PI/Hoechst staining (scale bar, 400 μm), (**D**) quantification of PI-positive area, (**E**) CPE (scale bar, 400 μm), and (**F**) LDH release in infected cells pretreated with KN-93 (10 μM). Data are presented as mean ± SD from three independent experiments. Statistical significance was determined using a two-tailed Student’s *t*-test (***P < 0.001).

CaMKII is a well-known downstream effector of Ca^2+^ signaling and has been implicated in cellular stress and injury (27, 37–39). We observed a time-dependent increase in CaMKII phosphorylation in hSD-1/2019-infected ARPE-19 cells (Fig. 5A). Pretreatment with the CaMKII inhibitor KN-93 reduced CaMKII phosphorylation in infected cells (Fig. 5B).

We next tested whether CaMKII inhibition affected the injury phenotype. Compared with infected cells without inhibitor treatment, KN-93-treated cells showed reduced PI staining, a lower PI-positive area, milder CPE, and decreased LDH release (Fig. 5C through F). Under comparable conditions, KN-93 did not markedly reduce viral genome accumulation or VP26-mCherry fluorescence (Fig. S1D through F). These results indicate that CaMKII activation participates in hSD-1/2019-induced retinal epithelial cell injury.

### Extracellular Ca^2+^ influx and CaMKII act upstream of JNK1/2 activation

**Fig 6:**
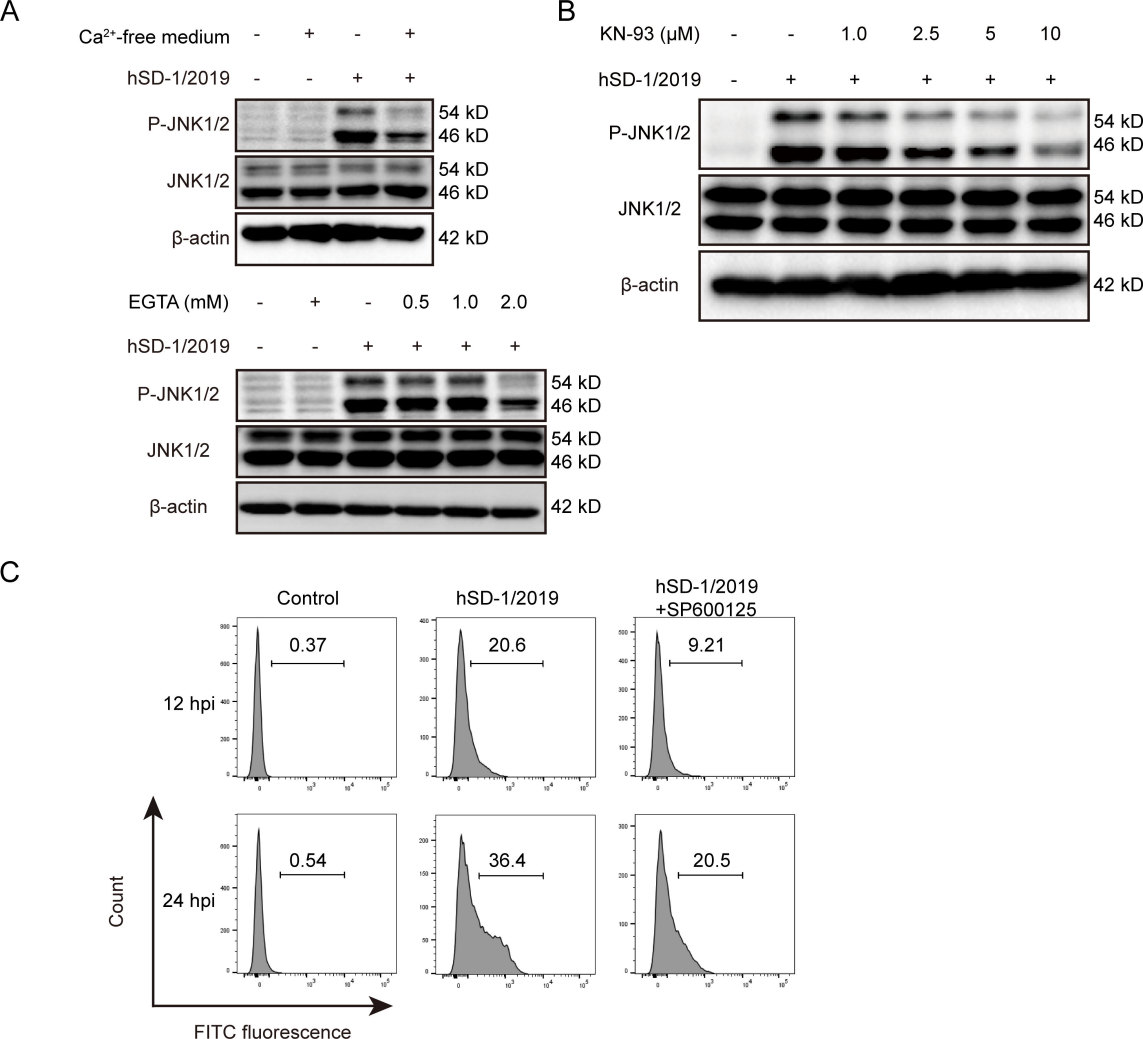
Extracellular Ca^2+^ influx and CaMKII act upstream of JNK1/2 activation. (**A**) Western blot analysis of phospho-JNK1/2 levels in ARPE-19 cells infected with hSD-1/2019 (MOI = 0.5) for 16 h, cultured in Ca^2+^-free medium or treated with EGTA at the indicated concentrations. Total JNK1/2 and β-actin were used as controls. (**B**) Western blot analysis of phospho-JNK1/2 levels in infected cells treated with KN-93 at the indicated concentrations. Total JNK1/2 and β-actin were used as controls. (**C**) [Ca^2+^]ᵢ in infected cells pretreated with the JNK1/2 inhibitor SP600125 (10 μM) was measured at 12 or 24 hpi by Fluo-3/AM staining and flow cytometry.

Having observed the involvement of extracellular Ca^2+^ influx, CaMKII, and JNK1/2 in hSD-1/2019-induced injury, we next examined their functional relationship. Blocking extracellular Ca^2+^ entry with Ca^2+^-free medium or EGTA markedly reduced JNK1/2 phosphorylation in hSD-1/2019-infected cells (Fig. 6A). Similarly, inhibition of CaMKII with KN-93 suppressed JNK1/2 phosphorylation in a dose-dependent manner, suggesting that CaMKII acts downstream of extracellular Ca^2+^ influx and upstream of JNK1/2 activation (Fig. 6B). SP600125 treatment also reduced sustained [Ca^2+^]ᵢ accumulation during infection, suggesting that JNK1/2 activity may contribute to sustained Ca^2+^ accumulation during infection (Fig. 6C).

### The Ca^2+^/CaMKII/JNK axis contributes to mitochondrial dysfunction in hSD-1/2019-infected cells

**Fig 7:**
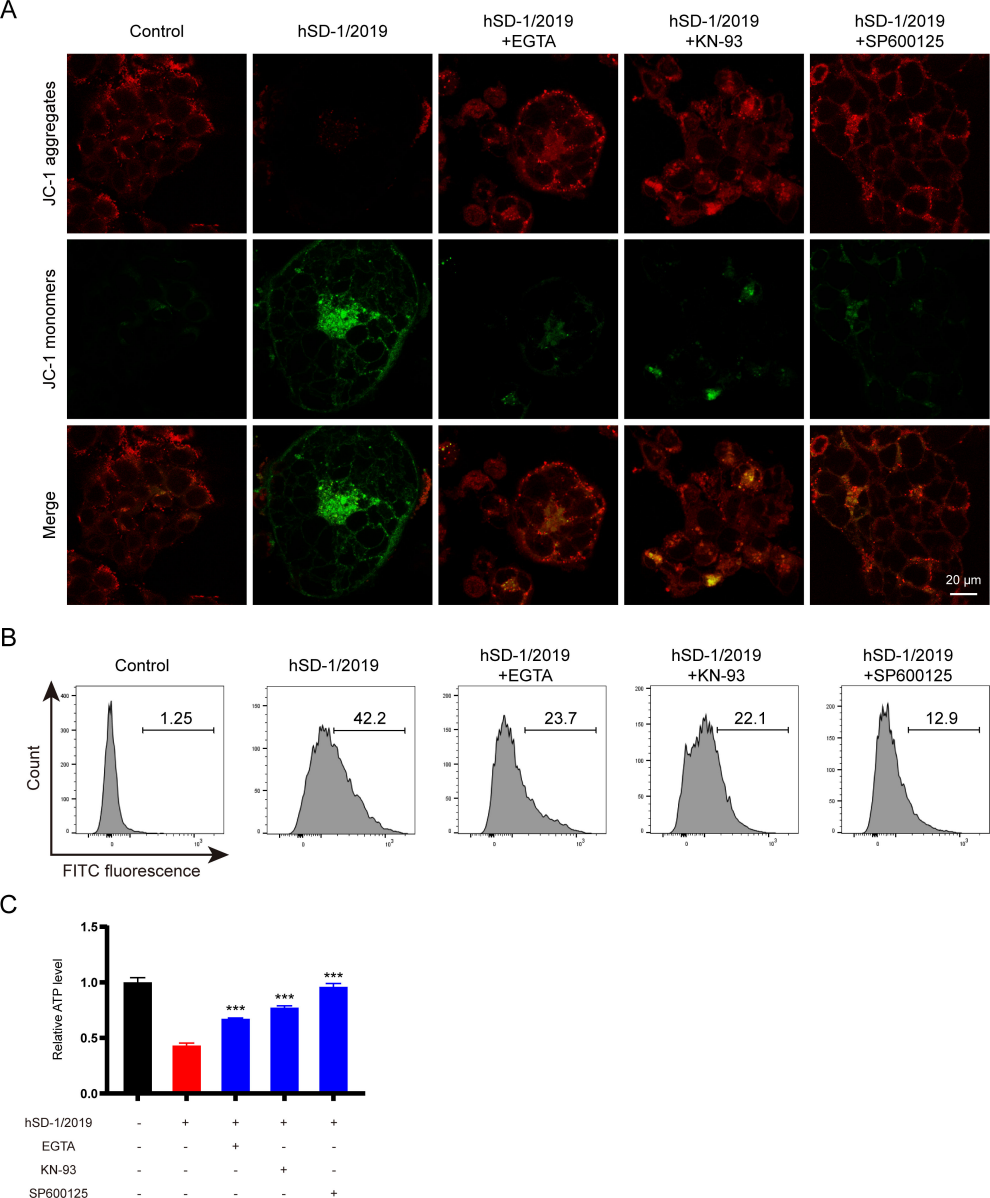
The Ca^2+^/CaMKII/JNK axis contributes to mitochondrial dysfunction in hSD-1/2019-infected cells. ARPE-19 cells were pretreated with EGTA (2 mM), KN-93 (10 μM), or SP600125 (10 μM) before infection with hSD-1/2019 (MOI = 0.5) for 16 h. (**A**) ΔΨm was assessed by JC-1 staining and confocal microscopy. A decrease in the red/green fluorescence ratio indicates ΔΨm loss. Scale bar, 20 μm. (**B**) Intracellular ROS levels were measured using DCFH-DA staining and flow cytometry. (**C**) Cellular ATP levels were quantified using an enhanced ATP assay kit. Data are presented as mean ± SD from three independent experiments. Statistical significance was determined using a two-tailed Student’s *t*-test (***P < 0.001).

We next examined whether the Ca^2+^/CaMKII/JNK signaling axis was linked to mitochondrial dysfunction in hSD-1/2019-infected ARPE-19 cells. JC-1 staining showed a marked loss of ΔΨm after infection, as reflected by reduced JC-1 aggregates and increased JC-1 monomers (Fig. 7A). Pretreatment with EGTA, KN-93, or SP600125 partially restored the JC-1 staining pattern, indicating attenuation of mitochondrial depolarization.

Consistently, hSD-1/2019 infection increased intracellular ROS levels, and this increase was reduced by EGTA, KN-93, or SP600125 treatment (Fig. 7B). Infection also decreased cellular ATP levels, whereas blockade of extracellular Ca^2+^ influx, CaMKII, or JNK1/2 partially restored ATP levels (Fig. 7C). Together, these results link hSD-1/2019-induced mitochondrial dysfunction to the Ca^2+^/CaMKII/JNK signaling axis.

### Cav2.2-sensitive Ca^2+^ influx links extracellular Ca^2+^ entry to JNK1/2 activation and cell injury

**Fig 8:**
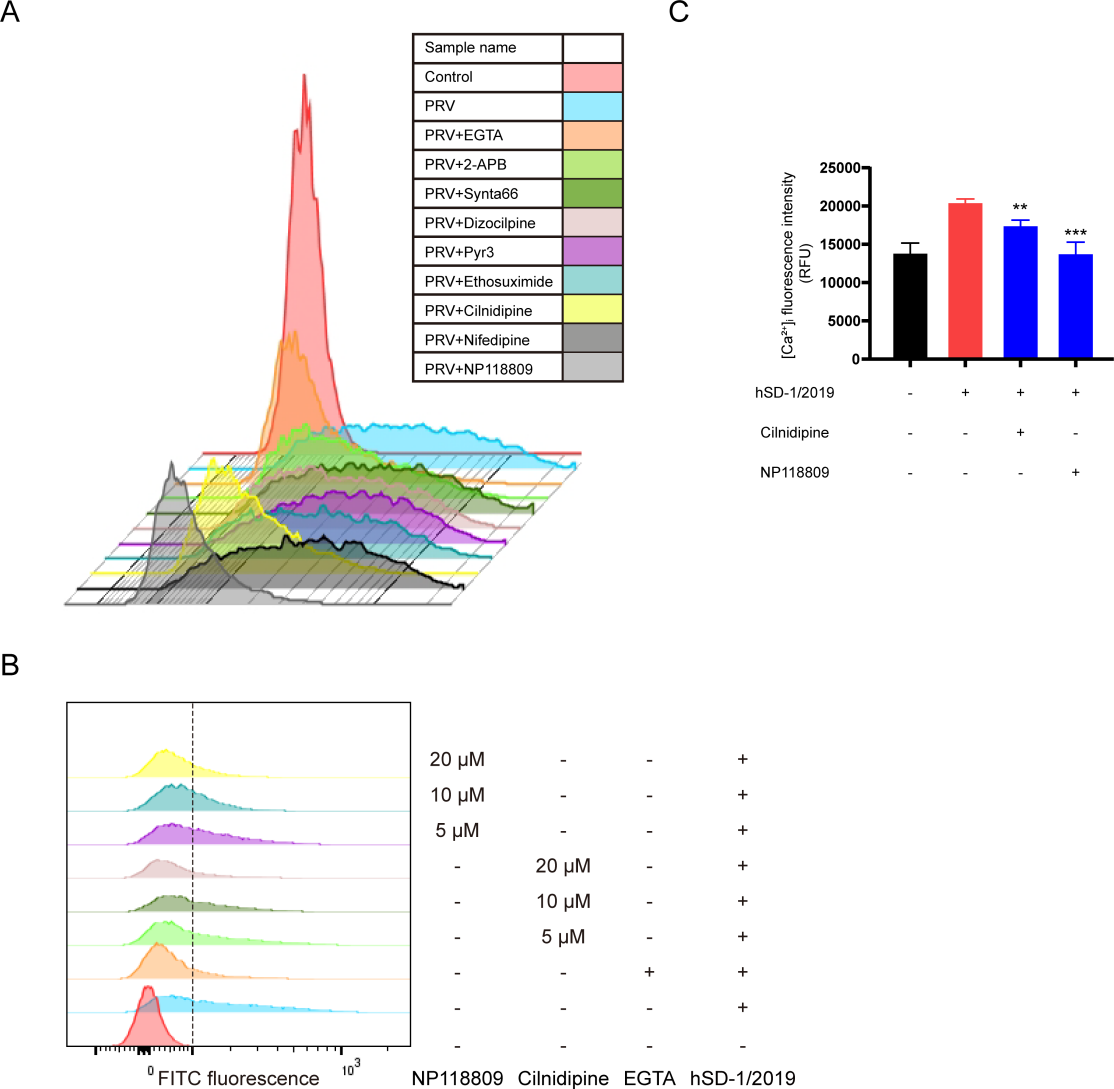
Pharmacological screening identifies a Cav2.2-sensitive component in hSD-1/2019-induced Ca^2+^ influx. ARPE-19 cells were infected with hSD-1/2019 (MOI = 0.5) for 16 h, and [Ca^2+^]ᵢ was measured by Fluo-3/AM staining and flow cytometry. (**A**) Screening of Ca^2+^ influx inhibitors affecting virus-induced [Ca^2+^]ᵢ elevation, including EGTA (extracellular Ca^2+^ chelator, 2 mM), 2-APB (SOCE inhibitor, 50 μM), Synta66 (Orai1 inhibitor, 10 μM), Dizocilpine (NMDA receptor antagonist, 10 μM), Pyr3 (TRPC3/ROC inhibitor, 10 μM), Ethosuximide (T-type voltage-gated Ca^2+^ channel inhibitor, 2 mM), Cilnidipine (L/N-type Ca^2+^ channel blocker, 20 μM), Nifedipine (L-type voltage-gated Ca^2+^ channel blocker, 10 μM), and NP118809 (N-type/Cav2.2 channel blocker, 20 μM). (**B**) Representative flow cytometry histograms showing dose-dependent effects of Cilnidipine or NP118809 on hSD-1/2019-induced [Ca^2+^]ᵢ elevation. EGTA (2 mM) was used as a positive control. (**C**) Quantification of [Ca^2+^]ᵢ in infected cells treated with Cilnidipine (20 μM) or NP118809 (20 μM). Data are presented as mean ± SD from three independent experiments. Statistical significance was determined using a two-tailed Student’s *t*-test (**P < 0.01; ***P < 0.001).

To identify the potential route of extracellular Ca^2+^ entry, we screened a panel of pharmacological inhibitors targeting major Ca^2+^ influx pathways. Among the compounds tested, Cilnidipine (an L/N-type channel blocker) and NP118809 (an N-type/Cav2.2 blocker) markedly attenuated the hSD-1/2019-induced increase in [Ca^2+^]ᵢ, whereas inhibitors targeting other Ca^2+^ entry routes showed weaker or limited effects (Fig. 8A). Dose-response analysis further showed that both Cilnidipine and NP118809 reduced the infection-associated increase in [Ca^2+^]ᵢ (Fig. 8B and C). These results pointed to a prominent Cav2.2-sensitive component in hSD-1/2019-induced Ca^2+^ influx.

**Fig 9:**
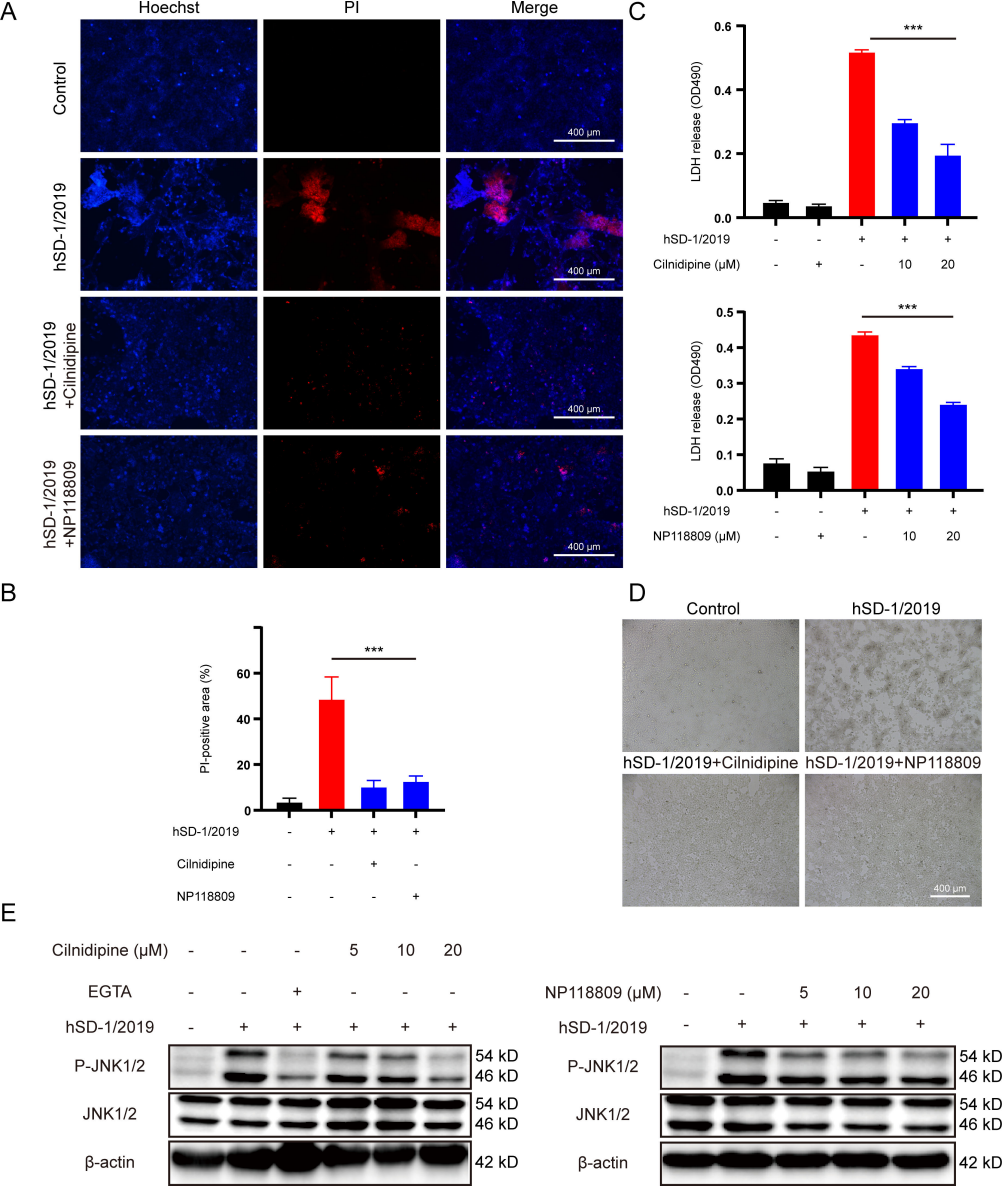
Cav2.2-sensitive channel blockade attenuates hSD-1/2019-induced cell injury and JNK1/2 activation. ARPE-19 cells were pretreated with Cilnidipine or NP118809 for 2 h before infection with hSD-1/2019 (MOI = 0.5) for 16 h. (**A**) PI/Hoechst staining of infected cells treated with Cilnidipine (20 μM) or NP118809 (20 μM). Scale bar, 400 μm. (**B**) Quantification of PI-positive area. (**C**) LDH release in infected cells treated with the indicated concentrations of Cilnidipine or NP118809. (**D**) CPE in infected cells treated with Cilnidipine (20 μM) or NP118809 (20 μM). Scale bar, 400 μm. (**E**) Western blot analysis of phospho-JNK1/2 levels in infected cells treated with the indicated concentrations of Cilnidipine or NP118809. EGTA (2 mM) was used as a positive control for extracellular Ca^2+^ restriction. Total JNK1/2 and β-actin were used as controls. Data are presented as mean ± SD from three independent experiments. Statistical significance was determined using a two-tailed Student’s *t*-test (***P < 0.001).

Pretreatment with either Cilnidipine or NP118809 reduced PI staining, PI-positive area, LDH release, and infection-induced CPE in hSD-1/2019-infected ARPE-19 cells (Fig. 9A through D). Furthermore, both inhibitors reduced JNK1/2 phosphorylation in a dose-dependent manner (Fig. 9E), linking Cav2.2-sensitive Ca^2+^ influx to downstream JNK1/2 activation. Importantly, neither Cilnidipine nor NP118809 significantly affected viral genome accumulation or VP26-mCherry fluorescence (Fig. S1G through I). Together, these findings support a role for Cav2.2-sensitive Ca^2+^ influx in hSD-1/2019-induced retinal epithelial cell injury and downstream JNK1/2 activation.

### gK expression promotes Cav2.2-sensitive Ca^2+^ elevation and JNK1/2 activation

**Fig 10:**
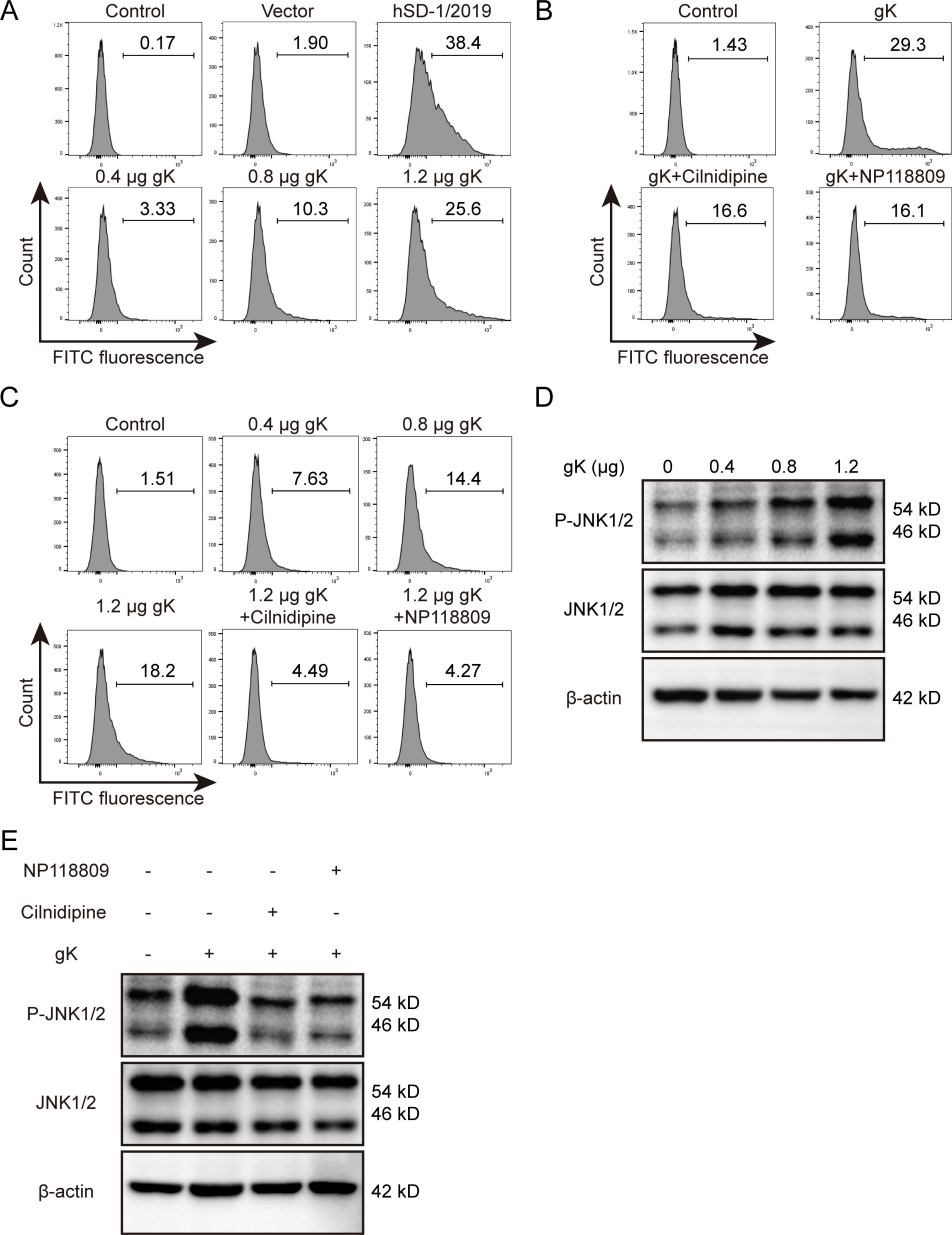
gK expression promotes Cav2.2-sensitive Ca^2+^ elevation and JNK1/2 activation. (**A**) [Ca^2+^]ᵢ in HEK-293T cells transfected with increasing amounts of gK-expressing plasmid or empty vector for 24 h, measured by Fluo-3/AM staining and flow cytometry. hSD-1/2019-infected HEK-293T cells were included as a reference. (**B**) [Ca^2+^]ᵢ in gK-transfected HEK-293T cells pretreated with Cilnidipine (20 μM) or NP118809 (20 μM). (**C**) [Ca^2+^]ᵢ in ARPE-19 cells transfected with increasing amounts of gK-expressing plasmid, with or without Cilnidipine (20 μM) or NP118809 (20 μM) treatment. (**D**) Western blot analysis of JNK1/2 phosphorylation in ARPE-19 cells transfected with increasing amounts of gK-expressing plasmid. (**E**) Western blot analysis of JNK1/2 phosphorylation in gK-transfected ARPE-19 cells treated with Cilnidipine (20 μM) or NP118809 (20 μM). Total JNK1/2 and β-actin were used as controls.

To explore potential viral contributors to the upstream Ca^2+^ signal, we first examined selected hSD-1/2019 proteins in a plasmid-expression system. Based on this initial candidate-protein analysis, gK was selected for further validation. Expression of gK induced a significant, dose-dependent increase in [Ca^2+^]ᵢ (Fig. 10A), and this gK-associated Ca^2+^ elevation was reduced by Cilnidipine or NP118809 treatment (Fig. 10B).

We next examined the same response in ARPE-19 cells. gK expression also increased [Ca^2+^]ᵢ in ARPE-19 cells in a dose-dependent manner, and the increase induced by gK was markedly reduced by Cilnidipine or NP118809 (Fig. 10C). In parallel, gK expression increased JNK1/2 phosphorylation in a dose-dependent manner in ARPE-19 cells (Fig. 10D). Moreover, Cilnidipine and NP118809 reduced gK-induced JNK1/2 phosphorylation (Fig. 10E). Together, these results indicate that gK expression can promote Cav2.2-sensitive Ca^2+^ elevation and JNK1/2 activation, supporting gK as a candidate upstream contributor to this signaling response during hSD-1/2019 infection.

## DISCUSSION

**Fig 11:**
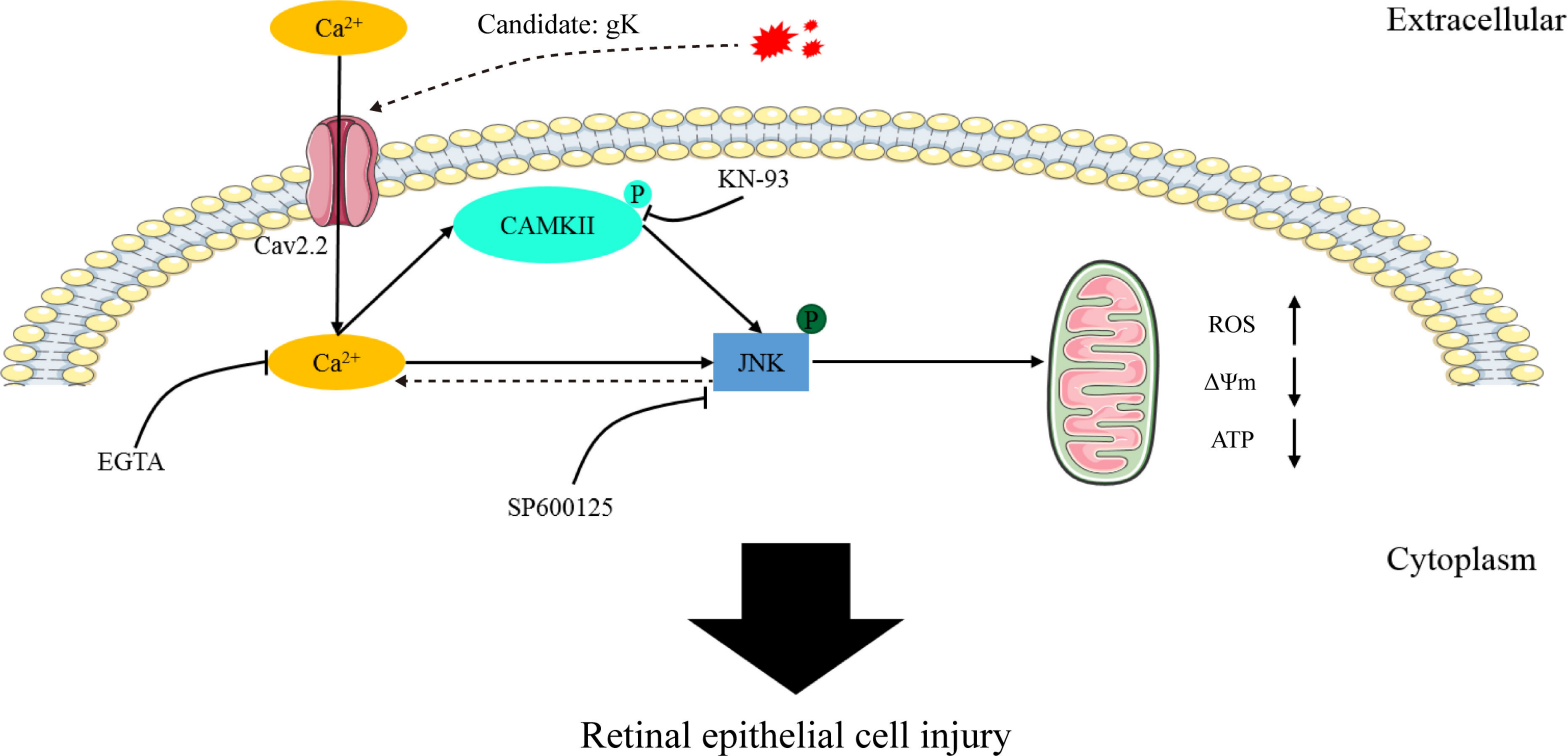
Proposed model of hSD-1/2019-induced retinal epithelial cell injury. hSD-1/2019 infection induces extracellular Ca^2+^ influx through a Cav2.2-sensitive pathway in ARPE-19 cells. The resulting increase in [Ca^2+^]ᵢ is associated with CaMKII activation and subsequent JNK1/2 phosphorylation. Activation of this signaling axis is linked to mitochondrial dysfunction, including increased ROS production, decreased ΔΨm, and ATP depletion, thereby contributing to retinal epithelial cell injury. PRV gK is proposed as a candidate viral contributor to the upstream Cav2.2-sensitive Ca^2+^ signal.

The present study identifies extracellular Ca^2+^ entry as a central event linking hSD-1/2019 infection to retinal epithelial injury in ARPE-19 cells. Although PRV was historically not regarded as a human pathogen (40), several reported human infections with severe ocular involvement have raised the question of how human-derived PRV isolates injure retinal cells (1, 6–8, 41). Our data suggest that hSD-1/2019-induced injury is not explained by infection or viral replication alone, but is closely associated with a pronounced extracellular Ca^2+^ influx that connects viral infection to stress kinase activation, mitochondrial dysfunction, and membrane-compromising cellular injury (Fig. 11). This framework places Ca^2+^ entry, particularly a Cav2.2-sensitive component, at the center of the retinal epithelial response to hSD-1/2019 infection.

Mechanistically, the Ca^2+^ response induced by hSD-1/2019 appears to involve more than one entry route. This is consistent with the broader concept that virus-associated ion flux may involve multiple host channels or viral membrane proteins, including viroporins (42–44). In our pharmacological screen, several inhibitors produced partial effects, whereas Cilnidipine and NP118809 showed the strongest and most consistent reduction in Ca^2+^ influx and associated injury readouts (Fig. 8A). These findings suggest that hSD-1/2019 infection does not merely disturb intracellular Ca^2+^ balance passively, but engages a Cav2.2-sensitive extracellular Ca^2+^ entry route that becomes functionally coupled to epithelial injury. A link between Ca^2+^ influx and virus-associated cytotoxicity has also been reported in dengue virus (DENV) studies, where DENV-induced cytotoxic factor triggered Ca^2+^ influx in susceptible immune cells (45). Although genetic validation will be required to define the precise contribution of Cav2.2, the pharmacological data identify this Ca^2+^ entry component as a prominent part of the hSD-1/2019-induced injury response.

Downstream of Ca^2+^ influx, the injury response appears to converge mainly on JNK1/2 activation and mitochondrial dysfunction. CaMKII may provide an upstream bridge between Ca^2+^ stress and JNK activation, consistent with previous work showing that CaMKII can connect Ca^2+^ dysregulation to JNK activation and loss of mitochondrial membrane potential (22). Similar stress-signaling connections have been described in other alphaherpesvirus infections, including HSV-induced ROS/MAPK activation and BHV-1-induced JNK activation in infected cells (46, 47). In our study, SP600125 attenuated mitochondrial depolarization, ROS accumulation, and ATP depletion, supporting JNK1/2 as a functional component of the mitochondrial injury response. Its effect on sustained [Ca^2+^]ᵢ further raises the possibility that JNK activity may help maintain late Ca^2+^ influx, in line with MAPK-dependent regulation of Ca^2+^ entry reported in neuronal injury models (48). However, whether such a feedback relationship operates during PRV infection in ARPE-19 cells remains to be defined.

The comparison with Ea is informative because it separates viral replication from injury-associated Ca^2+^ signaling. Ea increased viral genome copies in ARPE-19 cells (Fig. 1A), indicating that its limited Ca^2+^ response was not simply due to a failure of infection (Fig. S2A). However, Ea did not induce the strong EGTA-sensitive Ca^2+^ influx observed after hSD-1/2019 infection at the time points examined (Fig. S2B). This distinction suggests that extracellular Ca^2+^ entry is a defining feature of the hSD-1/2019 response in ARPE-19 cells, rather than a general consequence of PRV genome accumulation. It also points to the possibility that differences between hSD-1/2019 and Ea may lie not only in replication kinetics, but also in how viral membrane-associated functions engage host Ca^2+^ signaling.

Within this framework, gK provides a plausible viral entry point into the Ca^2+^-JNK injury axis. Studies of HSV-1 have shown that gK participates in virus-host membrane events, with the gK amino terminus required for Akt-dependent intracellular Ca^2+^ release (49). Our data extend this alphaherpesvirus gK-Ca^2+^ connection by showing that PRV gK expression induces Cav2.2-sensitive Ca^2+^ elevation and JNK1/2 activation in transfected cells, including ARPE-19 cells. However, gK should not be interpreted as the sole or established determinant of the hSD-1/2019 phenotype. Alphaherpesvirus entry, membrane fusion, and membrane-associated signaling are coordinated by multiple envelope proteins (50–52), and a similar multi-protein contribution may underlie the Ca^2+^ influx observed during hSD-1/2019 infection. Thus, gK is best viewed as a candidate upstream contributor in the hSD-1/2019 background. Direct gK-swap or chimeric-virus approaches will be needed to determine whether strain-specific gK function contributes to the different Ca^2+^ influx profiles of hSD-1/2019 and Ea.

Together, these findings support a model in which hSD-1/2019 converts infection into a Ca^2+^-influx-driven injury program in retinal epithelial cells. In this model, Cav2.2-sensitive extracellular Ca^2+^ entry acts upstream of JNK1/2 activation and mitochondrial dysfunction, while gK represents a candidate viral contributor that may link alphaherpesvirus membrane functions to this host injury response. Although ARPE-19 cells are widely used to study virus-retina interactions, they are an immortalized model and may not fully capture primary retinal physiology, particularly under subconfluent conditions (53). Further genetic validation and studies in primary retinal cells or *in vivo* models will therefore be required to define the contribution of Cav2.2 and gK more precisely. Nevertheless, this study provides a mechanistic framework for understanding how a human-derived PRV isolate engages host Ca^2+^ signaling to promote retinal epithelial injury.

## MATERIALS AND METHODS

### Cells, viruses, and plasmids

ARPE-19, HEK-293T, and porcine kidney cells (PK-15) were obtained from the American Type Culture Collection (ATCC, Manassas, VA, USA). ARPE-19, HEK-293T, and PK-15 cells were maintained in Dulbecco’s modified Eagle medium (DMEM; Gibco, Grand Island, NY, USA) supplemented with 10% fetal bovine serum (FBS; Gibco) at 37°C in a humidified atmosphere containing 5% CO₂.

The human-derived PRV isolate hSD-1/2019 (GenBank accession no. GCA_027938875.1) was previously isolated in our laboratory and described (54). The classical PRV strain Ea was also maintained in our laboratory. Virus stocks were propagated in PK-15 cells and titrated on PK-15 cells by TCID₅₀ assay. TCID₅₀ values were calculated using the Reed-Muench method. The hSD-1/2019-VP26-mCherry reporter virus was kindly provided by Dr. Huihui Guo and was generated as previously described (55). Briefly, mCherry was inserted in frame at the 3′ end of UL35, upstream of the UL35 terminator sequence, to generate a VP26-mCherry fusion protein, and recombinant viruses were purified by plaque isolation. This reporter virus was used to monitor infection-associated VP26-mCherry fluorescence in ARPE-19 cells.

Plasmids encoding individual hSD-1/2019 proteins were generated by cloning the corresponding coding sequences into the pCAGGS-HA expression vector and verified by Sanger sequencing. The hSD-1/2019 gK-expressing pCAGGS-HA plasmid was used for gK expression assays in HEK-293T and ARPE-19 cells.

### Antibodies, inhibitors, and reagents

The following primary antibodies were used: rabbit anti-JNK1/2, rabbit anti-phospho-JNK1/2 (Thr183/Tyr185), rabbit anti-p38, rabbit anti-phospho-p38 (Thr180/Tyr182), rabbit anti-ERK1/2, rabbit anti-phospho-ERK1/2 (Thr202/Tyr204), rabbit anti-phospho-CaMKII (Thr286), and rabbit anti-β-actin (all from Cell Signaling Technology, Danvers, MA, USA). HRP-conjugated goat anti-rabbit IgG secondary antibody was obtained from Boster Bioengineering (Wuhan, China).

Pharmacological inhibitors included SP600125, a JNK1/2 inhibitor; SB203580, a p38 inhibitor; KN-93, a CaMKII inhibitor; Cilnidipine, an L/N-type Ca^2+^ channel blocker; Nifedipine, an L-type voltage-gated Ca^2+^ channel blocker; NP118809, an N-type/Cav2.2 channel blocker; 2-APB, a commonly used store-operated Ca^2+^ entry inhibitor; Synta66, an Orai1/SOCE inhibitor; Dizocilpine, an NMDA receptor antagonist; Pyr3, a TRPC3 inhibitor used to probe receptor-operated Ca^2+^ entry; and Ethosuximide, a T-type voltage-gated Ca^2+^ channel inhibitor. All inhibitors were purchased from MedChemExpress (Monmouth Junction, NJ, USA).

Other reagents included EGTA, BAPTA-AM, PI, Hoechst 33342, Fluo-3/AM, ROS Assay Kit, JC-1 Mitochondrial Membrane Potential Detection Kit, and Enhanced ATP Assay Kit (Beyotime Biotechnology, Shanghai, China). Calcium-free medium was commercial calcium-free DMEM (DMEM, high glucose, no glutamine, no calcium; Gibco/Thermo Fisher Scientific, catalog no. 21068028).

### Virus infection and pharmacological treatment

For infection experiments, ARPE-19 cells were used as subconfluent monolayers at 80%–90% confluence. Unless otherwise indicated, cells were infected with PRV at an MOI of 0.5. After adsorption for 1 h at 37°C, the inoculum was removed, and cells were washed with PBS and incubated in fresh DMEM containing 2% FBS. All comparative experiments were performed under the same infection and culture conditions unless otherwise specified.

For inhibitor studies, cells were pretreated with the indicated concentrations of inhibitors for 2 h before infection. The compounds were maintained in the culture medium during viral adsorption and subsequent incubation unless otherwise stated. For extracellular Ca^2+^ depletion experiments, cells were treated with EGTA or cultured in commercial calcium-free DMEM under the indicated conditions. For infection-control experiments under inhibitor or Ca^2+^-modulating conditions, viral genome accumulation and VP26-mCherry fluorescence were assessed under the same pretreatment and infection conditions used in the corresponding injury assays.

### Viral genome quantification and VP26-mCherry fluorescence measurement

For viral genome quantification, infected cells and culture supernatants were collected together at the indicated time points and subjected to three freeze-thaw cycles. Viral DNA was extracted using the TIANamp Genomic DNA Kit (TIANGEN, Beijing, China). PRV genome copies were quantified by TaqMan qPCR targeting the gE gene as previously described (54). Absolute viral genome copy numbers were calculated using a recombinant plasmid standard curve.

To monitor infection-associated fluorescence, ARPE-19 cells were infected with hSD-1/2019-VP26-mCherry under the indicated conditions. Representative fluorescence images were acquired using a fluorescence microscope under identical exposure settings within each experiment. VP26-mCherry fluorescence intensity was quantified using a microplate reader at the indicated time points.

### Plasmid transfection and gK expression assays

For the candidate viral protein analysis, selected hSD-1/2019 open reading frames were cloned into the pCAGGS-HA expression vector. The candidates included representative envelope/membrane-associated proteins and infection-related proteins, such as UL20, UL49.5, UL51, UL53 (gK), US4, US8, UL13, UL41, UL48, and UL50. HEK-293T cells were transfected with the indicated plasmids using jetPRIME transfection reagent (Polyplus-transfection, Illkirch, France) according to the manufacturer’s instructions. At 24 h post-transfection, cells were loaded with Fluo-3/AM and analyzed by flow cytometry to assess [Ca^2+^]ᵢ. Based on this initial analysis, gK was selected for further validation.

For gK validation assays, HEK-293T cells were transfected with empty pCAGGS-HA vector or hSD-1/2019 gK-expressing pCAGGS-HA plasmid using jetPRIME transfection reagent. ARPE-19 cells were transfected using Lipofectamine 3000 reagent (Thermo Fisher Scientific) according to the manufacturer’s instructions. For dose-response experiments, cells were transfected with increasing amounts of gK-expressing plasmid as indicated. At 6 h post-transfection, the transfection medium was replaced with fresh culture medium, with Cilnidipine (20 μM) or NP118809 (20 μM) added where indicated. Cells were collected at 24 h post-transfection for [Ca^2+^]ᵢ measurement or western blot analysis.

### PI/Hoechst staining and quantification of PI-positive area

Membrane permeability was assessed by PI/Hoechst 33342 co-staining. Briefly, cells were washed with PBS and incubated with PI (2 μg/mL) and Hoechst 33342 according to the manufacturer’s instructions for 15 min at room temperature in the dark. Fluorescence images were acquired using a Leica fluorescence microscope under identical exposure settings within each experiment.

PI-positive area was quantified using ImageJ. The same threshold settings were applied to images within each experiment, and the PI-positive area was normalized to the total analyzed field area. At least three randomly selected fields were analyzed for each condition in each independent experiment.

### LDH release assay

Cytotoxicity was quantified by measuring LDH release into the culture supernatant using an LDH Cytotoxicity Assay Kit (Beyotime) according to the manufacturer’s instructions. Absorbance was measured at 490 nm using a microplate reader. LDH release was presented as OD490 values unless otherwise indicated.

### Annexin V/PI flow cytometry

Infected cells were harvested at the indicated time points, washed twice with cold PBS, and stained with Annexin V-FITC and PI (Beyotime) according to the manufacturer’s instructions. Samples were analyzed immediately by flow cytometry. Uninfected and single-stained controls were used for compensation and gating. Unless otherwise indicated, flow cytometry data were analyzed using FlowJo v10 software (BD Biosciences, Ashland, OR, USA).

### Phase-contrast microscopy and TEM

Cell morphology and CPE were observed using a phase-contrast inverted microscope (Leica, Germany). Images were acquired under comparable settings within each experiment.

For TEM, cells were fixed in 2.5% glutaraldehyde, post-fixed in 1% osmium tetroxide, dehydrated through a graded ethanol series, and embedded in epoxy resin. Ultrathin sections were stained with uranyl acetate and lead citrate and examined using a Tecnai G2 20 TWIN transmission electron microscope (FEI, USA).

### Intracellular Ca^2+^ measurement

[Ca^2+^]ᵢ was measured using Fluo-3/AM. Cells were loaded with Fluo-3/AM (5 μM) for 30 min at 37°C, washed with PBS, and analyzed by flow cytometry. For endpoint measurements in 96-well plates, Fluo-3/AM fluorescence intensity was measured using a microplate reader as an overall readout of [Ca^2+^]ᵢ. The same acquisition settings were used within each experiment.

### Western blot analysis

Cells were lysed in RIPA buffer containing protease and phosphatase inhibitors (Beyotime). Protein concentrations were determined using a BCA Protein Assay Kit (Beyotime). Equal amounts of protein were separated by SDS-PAGE and transferred onto PVDF membranes. Membranes were blocked with 5% BSA in TBST for 1 h at room temperature and incubated with primary antibodies overnight at 4°C, followed by HRP-conjugated secondary antibodies. Protein signals were detected using an enhanced chemiluminescence kit (Bio-Rad, USA) and imaged with a ChemiDoc XRS+ system (Bio-Rad, USA).

### Mitochondrial function assays

ΔΨm was assessed using a JC-1 Mitochondrial Membrane Potential Detection Kit (Beyotime). Cells were incubated with JC-1 for 20 min at 37°C. The red (J-aggregates) and green (J-monomers) fluorescence signals were detected by confocal microscopy, with emission measured at approximately 590 nm (aggregates) and 530 nm (monomers). The red/green fluorescence ratio was used as an indicator of ΔΨm.

Intracellular ROS levels were measured using DCFH-DA. Cells were incubated with DCFH-DA (5 μM) for 20 min at 37°C, and fluorescence intensity was analyzed by flow cytometry.

Intracellular ATP levels were measured using an Enhanced ATP Assay Kit (Beyotime), and luminescence was recorded using a microplate reader.

### Statistical analysis

All experiments were performed with at least three biological replicates unless otherwise indicated. Data are presented as mean ± standard deviation (SD). Statistical analyses were conducted using GraphPad Prism 8.0. Two-group comparisons were analyzed using a two-tailed Student’s *t*-test. Multiple-group comparisons were analyzed using one-way or two-way ANOVA, followed by Dunnett’s or Tukey’s post hoc test where appropriate. A *P* value < 0.05 was considered statistically significant.

## Data Availability

All data supporting the findings of this study are available within the article and its supplemental material.

## SUPPLEMENTAL MATERIAL

Figures S1 and S2 spectrum.01655-26-s0001.pdf Infection-related readouts under pharmacological or Ca²⁺-modulating conditions and the Ca²⁺ response induced by the classical PRV strain Ea in ARPE-19 cells.

ASM does not own the copyrights to Supplemental Material that may be linked to, or accessed through, an article. The authors have granted ASM a non-exclusive, world-wide license to publish the Supplemental Material files. Please contact the corresponding author directly for reuse.
