# Supplementary material for: A human-derived Pseudorabies virus isolate induces Cav2.2-sensitive Ca2+ influx and JNK-associated retinal epithelial injury
Source: Microbiol Spectr. 2026 Jul 16;14(8):e01655-26. doi: 10.1128/spectrum.01655-26 (PMC13436308; doi:10.1128/spectrum.01655-26)
Supplement: Supplementary file 1 [file spectrum.01655-26-s0001.pdf]

Supplementary Materials

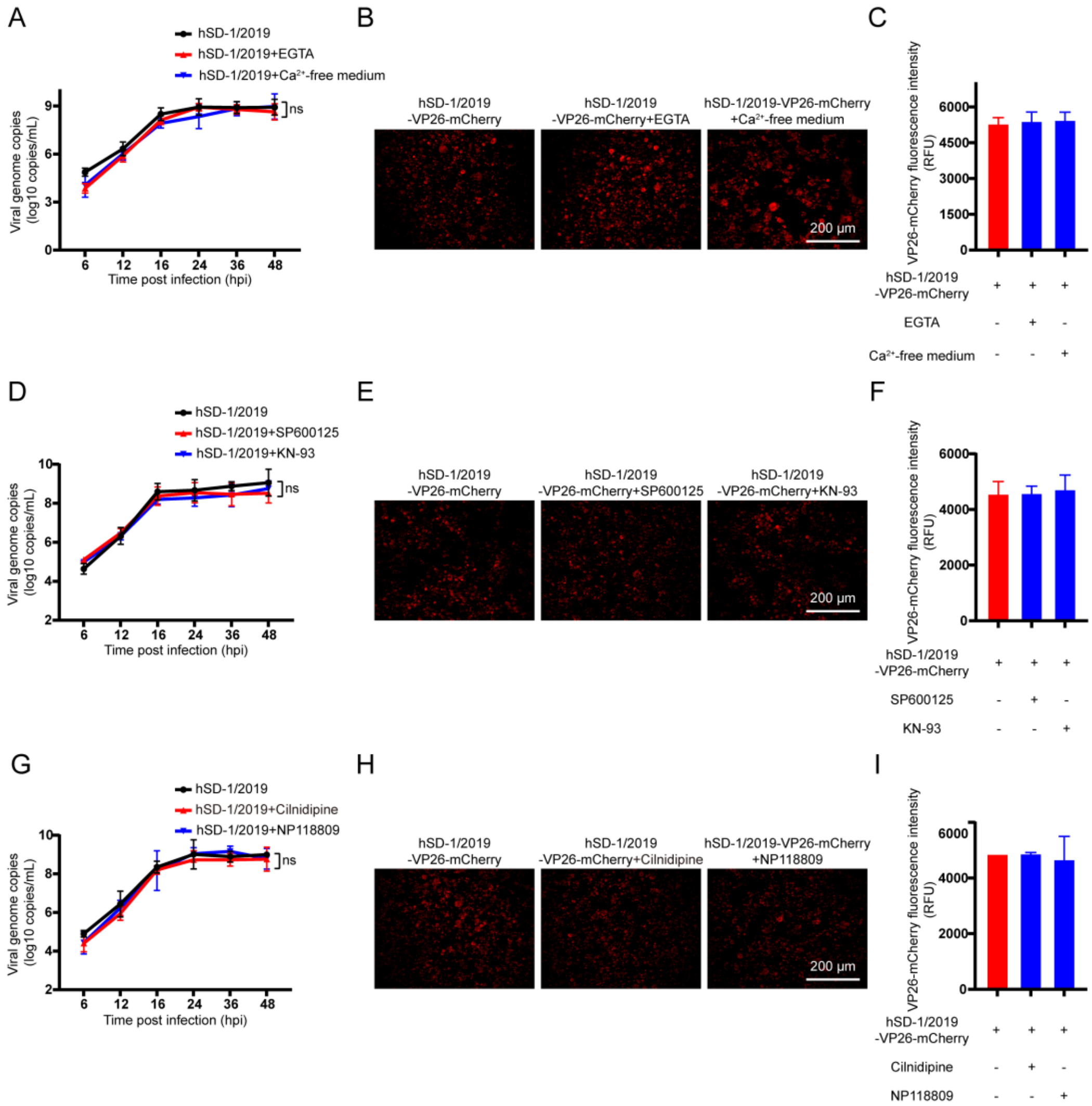

Figure S1. Effects of pharmacological inhibitors on hSD-1/2019 infection-related readouts in ARPE-19 cells.

ARPE-19 cells were pretreated with the indicated inhibitors for 2 h before infection with hSD-1/2019 or hSD-1/2019-VP26-mCherry at MOI = 0.5. (A, D, G) Viral genome accumulation in hSD-1/2019-infected ARPE-19 cells under the indicated treatment conditions. Total viral genome copies were quantified by qPCR at the indicated time points post infection. (B, E, H) Representative fluorescence microscopy images showing VP26-mCherry signal in infected ARPE-19 cells under the indicated treatment conditions. Scale bar, 200 μm. (C, F, I) Quantification of VP26-mCherry fluorescence

intensity in infected ARPE-19 cells. Panels A-C show EGTA (2 mM) and Ca^{2+}-free medium; panels D-F show SP600125 (10 μM) and KN-93 (10 μM); panels G-I show Cilnidipine (20 μM) and NP118809 (20 μM). Data are presented as mean ± SD from three independent experiments. Statistical significance was determined using a two-tailed Student's t-test (ns, not significant).

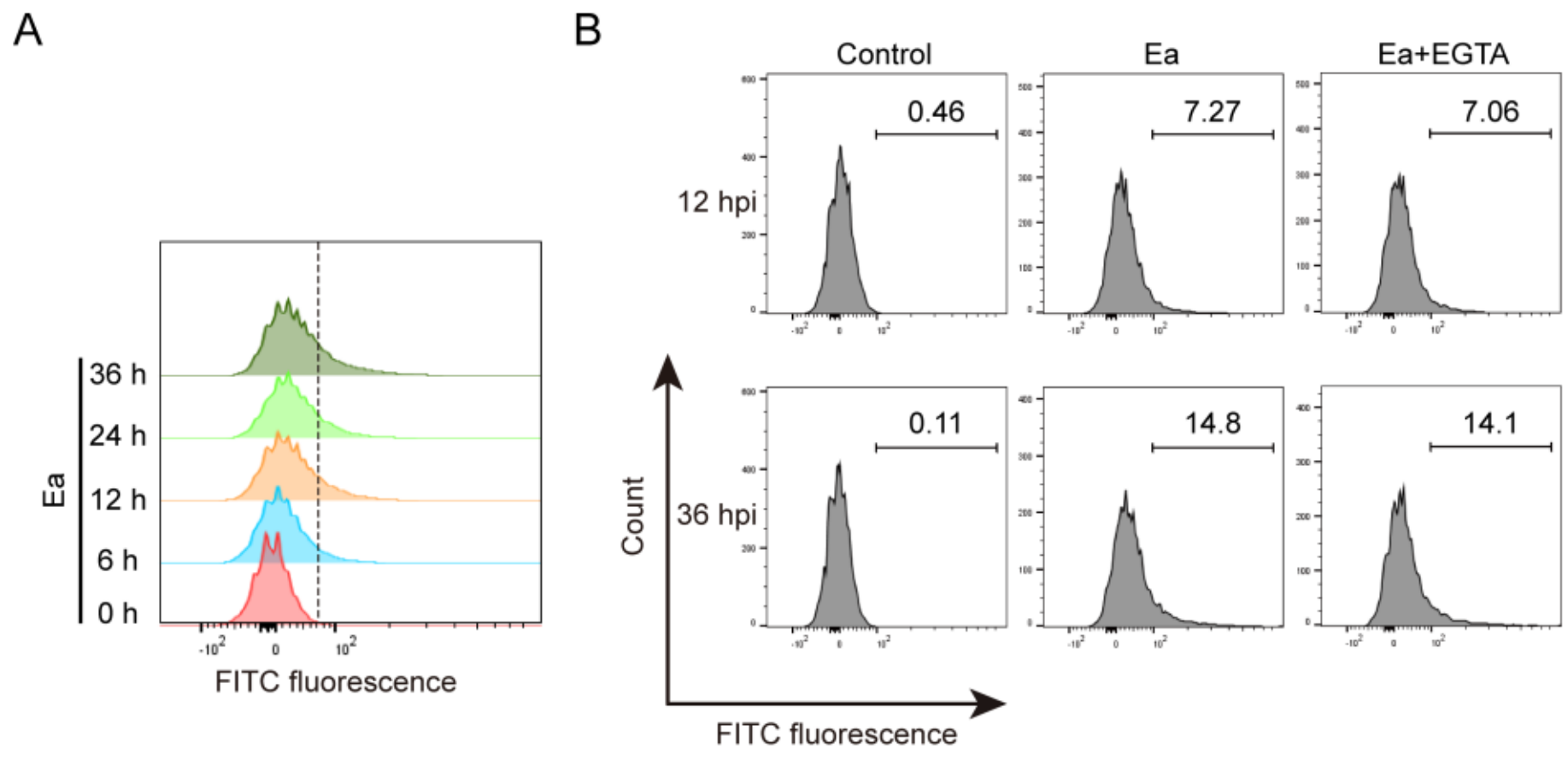

Figure S2. The classical PRV strain Ea induces a limited Ca^{2+} response in ARPE-19 cells.

ARPE-19 cells were infected with the classical PRV strain Ea at MOI = 0.5, and $[Ca^{2+}]_i$ was measured by Fluo-3/AM staining and flow cytometry. (A) Time-course analysis of $[Ca^{2+}]_i$ in Ea-infected ARPE-19 cells at the indicated times post infection. (B) $[Ca^{2+}]_i$ in control and Ea-infected cells in the presence or absence of EGTA (2 mM) at 12 or 36 hpi. The numbers in the flow cytometry plots indicate the percentage of cells within the indicated fluorescence gate.
